# Inflammation identity crisis: pathogenic nature of platelet-derived transforming growth factor β in chronic inflammatory conditions

**DOI:** 10.3389/fimmu.2026.1938898

**Published:** 2026-10-05

**Authors:** Karli R. Sutton, Rachel C. Eisenhart, Meera V. Singh

**Affiliations:** 1 Department of Immunology and Microbiology, University of Rochester School of Medicine and Dentistry, Rochester, NY, United States; 2 Department of Neurology, University of Rochester School of Medicine and Dentistry, Rochester, NY, United States; 3 Department of Neuroscience, University of Rochester School of Medicine and Dentistry, Rochester, NY, United States

**Keywords:** chronic inflammation, fibrosis, platelets, TGFβ, vascular disease

## Abstract

Chronic inflammatory conditions are associated with sustained platelet activation and subsequent release of platelet-derived immunomodulatory factors. Among these, platelets are the largest circulating reservoir of transforming growth factor β (TGFβ), and as such can be implicated in all physiological processes where TGFβ plays a crucial role. TGFβ is associated with immune regulation via dampening of Th_1_ responses, amplification of regulatory T lymphocyte polarization, and reduced inflammation, which can be beneficial in mitigating autoimmune diseases. However, in the case of solid tumors or persistent viral infections, this function can result in aberrant immune suppression and increased disease severity and pathology. TGFβ is also implicated in tissue remodeling and wound healing, however, in conditions associated with chronic platelet activation, such as diabetes, hypertension, and HIV infection, increased levels of platelet-derived TGFβ can exacerbate fibrosis, resulting in tissue and organ damage. Thus, whether TGFβ plays a pathogenic or pro-homeostatic role is dependent on the disease context. In order to harness the therapeutic potential of platelet-derived TGFβ, it is crucial to first understand how its mechanism of action varies across physiologic and pathologic conditions. In this review, we aim to emphasize the highly pleiotropic nature of TGFβ, highlight what is currently known about platelet-derived TGFβ across disease states, and to reinforce the importance of considering platelet-derived mediators as regulators of immunity and tissue responses, especially in the presence of chronic inflammation.

## 1 Introduction

Involved in the maintenance of homeostatic regulation and disease response, transforming growth factor-β (TGFβ) is a key cytokine associated with a wide variety of physiologic and immunologic processes. TGFβ in the broadest sense refers to a superfamily of proteins, including several activins, growth differentiation factors, Nodal, and associated antagonistic ligands (1). We will be focusing on the first and most ubiquitously expressed TGFβ isoform, TGFβ1 which will simply be notated as TGFβ unless otherwise specified throughout the remainder of this review. Following injury, TGFβ responds to changes in the ECM, where it is known to promote the proliferation and differentiation of stem cells and partake in wound repair. TGFβ acts as a critical recruiter of osteogenic bone marrow mesenchymal stem cells during the bone remodeling process, while simultaneously functioning as an immunomodulator to recruit other effector cells to the injury site (2). TGFβ can promote angiogenesis through the production of vascular endothelial growth factor A, and it’s overexpression can be a powerful driver of fibrosis (3–6). This effect is seen especially in cancers, where distinct populations of cancer-associated fibroblasts are characterized by a “TGF-β-driven matrix-producing contractile phenotype” (1). Lastly, TGFβ is known to regulate immunosuppressive function of immune cells (7–12). TGFβ has been shown to be an important factor in the acceptance and tolerance of stem cell transplantation, polarization of myeloid derived suppressor cells (MDSCs), and suppression of autoimmunity via regulatory T cell interaction with these MDSCs (10, 13–16). TGFβ has also been implicated in reducing immune activity in various cancer types due to its regulatory and suppressive nature (17–19).

TGFβ is synthesized as a disulfide linked dimeric precursor (20). This is later cleaved in the Golgi by furin, therefore converting it to the small latent TGFβ complex (SLC), with the latency associated peptide (LAP) on the N-terminus and the mature TGFβ cytokine on the C-terminus (1). Before its secretion, the SLC is bonded to latent TGFβ-binding protein (LTBP1) or Glycoprotein A repetitions predominant protein (GARP), especially in platelets and regulatory T cells (21, 22) via disulfide linkages forming the large latent complex (LLC). Regardless of origin, TGFβ must be first be converted into its biologically active form, with different cell types achieving this through varying mechanisms. Integrins, αvβ8 and αvβ6 have been found to play a crucial role in this process (1). Integrin αvβ6, which is most readily expressed by epithelial cells, activates latent TGFβ through a physical deformation of the linkage between TGFβ and the LAP, which occurs when cells expressing αvβ6 bind to the RGD sequence of the LAP and are induced to contract (1). Upon this contraction, mechanical forces pull the mature TGFβ apart from the LAP, exposing its TGFβ receptor binding domains. Actin-myosin contraction is also involved in this process (23). Integrin αvβ8, has been implicated in TGFβ activation, especially in monocytes. Monocytes engage in both paracrine and autocrine TGFβ signaling by activating endogenous and exogenous TGFβ (24–26). Recent work has shown that activation through αvβ8 may function by inducing a conformational change in the LAP that allows receptor binding without complete dissociation of the LAP from the mature TGFβ (1). While, integrin αvβ8 is readily expressed by all monocyte subpopulations (classical, intermediate, and non-classical), classical monocytes were found to be most efficient at activating TGFβ in a synergistic manner by integrin αvβ8 and matrix metalloproteinase 14 (MMP14) (24). TGFβ is also known to be activated by other cell types via proteases including plasmin, cathepsin and matrix metalloproteinase 9 (MMP9), which cleave the LAP, therefore releasing activated TGFβ (27, 28). Recent cryoEM studies have uncovered that LLC has a domain swapped architecture, where the straitjacket and arm domains are either linked in an ipsilateral or a contralateral conformation. The ipsilateral arrangement requires complete dissociation of the latent complex by integrins, while the contralateral conformation can be activated without complete release from the covalently bound LAP (29, 30). Once activated, TGFβ binds to the TGF receptor complex, which is expressed on the cell membranes of various cell types, kicking off the canonical and non-canonical signaling cascade (27). The signal transduction of TGFβ is well characterized and relies on a host of SMAD transcription factors (1).

Almost all cell types can produce TGFβ but within the extracellular matrix (ECM), fibroblasts, endothelial, epithelial cells and osteoclasts contribute to its localized deposition (31–33). On the other hand, in circulation, platelets are a grossly overlooked, but main producer of TGFβ, containing over 40–100 times more TGFβ than any other cells, and accounting for about 40-90% of plasma TGFβ (34–36). While there are published reports discussing the role of TGFβ in health and disease, it is crucial to understand how platelet derived TGFβ contributes to the immunopathogenesis of chronic inflammatory conditions associated with persistent platelet activation and that is the subject of this review. All crucial studies which have focused on platelet-derived TGFβ are summarized in **Supplementary Table 1**.

## 2 Platelets in immunity and chronic inflammation

### 2.1 Platelets as immune cells

It has long been established that platelets are crucial during thrombosis and wound healing, but over the last decade, these cells have emerged as key players in the greater immune milieu. Platelets are fragments of their progenitor cells, megakaryocytes and are prepackaged with alpha- and dense- granules (α-granules and δ-granules, respectively). α-granules contain a wide variety of multifunctional coagulation factors, key adhesion molecules, cytokines, chemokines, immune modulators, and an array of complement and growth factors, while δ-granules contain chemical stimuli necessary for productive wound healing such as Ca^2+^, ADP, ATP and serotonin (37–43). A key feature of platelets is their propensity to become activated under conditions of inflammation or injury which canonically results in the commencement of the coagulation cascade. Two main events occur during platelet activation: adhesion and degranulation. Adhesion to extracellular matrices (ECM) is the initial step in the platelet activation pathway and hemostasis (44–47). Crosslinking of collagen and platelet glycoproteins, specifically GP VI, results in degranulation of α- and δ-granules (38–40, 44, 48, 49). As mentioned previously, these granules house key molecules necessary for the continuation and amplification of the coagulation cascade (δ-granules) and for cellular communication and immune modulation (α-granules) (37–43).

It is important to note that α-granules are found at a frequency of between 50 to 80 α-granules per platelet, which is about 10 times more than δ-granules (37, 50). This means that each platelet is highly concentrated with immune factors, cytokines, chemokines, and adhesion molecules. For example, platelet α-granules contain high concentrations of CD40L (CD154) (51–53). CD40L is a member of the tumor necrosis factor (TNF) super family and is a key mediator in lymphocyte regulation, as well as endothelial cell stimulation (51–55). Platelet-derived CD40L has been linked to a broad range of innate and adaptive immune events including, but not limited to enhanced recruitment of immune cells, dendritic cell maturation, and has been implicated in atherosclerosis pathology (54, 56–60). Additionally, α-granules contain large amounts of platelet factor 4, also known as CXCL4. CXCL4 binds to the surface of monocytes and subsequently facilitates endothelial migration to the site of inflammation or infection, inhibits apoptosis, and drives monocyte differentiation into macrophages (61–63). Interleukin 1 (IL-1) is another α-granule housed cytokine that is directly involved in inflammatory modulation. Specifically, IL-1β induces cytokine production in vascular smooth muscle cells which is directly tied to vascular pathogenesis, like that found in atherosclerosis (64, 65).

As is the focus of this review, platelets are considered to be the main producer of TGFβ in the body, containing over 40 times more TGFβ than other cells, and accounting for over 40 to 90% of plasma TGFβ levels (34–36). Perturbations of TGFβ signaling have been associated with decreased self-tolerance in immune thrombocytopenia due to severely reduced serum TGFβ levels (66–69). In this regard, studies from the Morrell group have shown that platelet-derived TGFβ and β2M are responsible for regulating monocyte polarization following acute vascular injury (70, 71). Under normal conditions, platelets secrete homeostatic levels of TGFβ that bind to the TGFβ receptor on the surface of monocytes that then signal through the canonical SMAD2/3 cascade and result in an increase in pro-reparative transcripts (70). Following vascular injury, the levels of pro-inflammatory, platelet-derived β2M rise and cross a threshold of 2 µg/mL which then outcompetes TGFβ for the binding site on the TGFβ receptor (71). β2M-predominant signaling leads to a SMAD independent, non-canonical signaling that results in increased NFκB and JNK transcript trafficking and subsequent proinflammatory polarization of monocytes (70, 71). These reports by the Morrell group are the first to implicate the β2M-TGFβR1 competitive binding axis in monocyte polarization during acute vascular events using mouse models. Further investigations into whether this signaling cascade occurs in humans during chronic inflammatory conditions (where β2M concentrations may never cross the threshold required to induce β2M-predominant signaling) need to be conducted.

### 2.2 Platelet activation in chronic inflammatory conditions

Multiple different health conditions are associated with aberrant platelet activation, albeit at levels lower than an acute vascular event such as a stroke or a myocardial infarction. For example, age related comorbidities such as diabetes, obesity, and hypertension (72–80). There is well documented evidence that hyperglycemia is directly related to an increase in vascular and ischemic events (81–88). People living with diabetes often exhibit platelet hyperreactivity at baseline when compared to people without diabetes (89–91). Increased platelet activation, and as a consequence, the higher risk of clot formation, is one of the primary reasons that each of these conditions share an elevated risk of cardiovascular disease (CVD) and thrombotic events such as stroke or heart attack (92–97).

Both small and large vessel diseases are associated with an increased level of platelet activation. Most notably, platelets are crucial for the initiation and progression of plaque formation in atherosclerosis (98, 99). Atherosclerosis (AS) is a vascular disease commonly associated with large arteries wherein plaques consisting of fats, cholesterol, and various cell types build within and around the arterial walls (100, 101). The initial trigger for plaque formation is suspected to be an injury to the vasculature that exposes components of the ECM, thus triggering platelet adherence and degranulation (98, 99, 102). Platelet degranulation and subsequent cytokine and chemokine release then recruits other immune cells to the cite of the plaque, tethering them to the endothelium, which then results in increased disease pathology and severity of the plaque (103–107). Other examples of large-vessel diseases include both coronary and peripheral artery diseases which have been shown to be exacerbated by increased platelet activation (108–112).

Although not as well characterized as large-vessel disease, there is evidence that platelet activation plays a role in the progression of small-vessel disease. Cerebral small vessel disease (CSVD) is a condition defined by increased stroke risk and observed cognitive decline (113). The key features of CSVD include damage to the small blood vessels in the brain due to inflammation, blood brain barrier dysfunction, hardening of the arteries, plaque accumulation, or vessel wall thickening (114). Although age is the primary factor behind CSVD progression, conditions such as kidney disease, hypertension, high cholesterol, and diabetes, lifestyle choices like smoking, and genetic factors all contribute to the risk of CSVD (114, 115). CSVD, much like AS, is associated with increased levels of platelet activation and platelet-monocyte complex (PMC) formation (116–118). PMC formation pushes monocytes towards a proinflammatory phenotype which is known to be involved in atherosclerotic progression (118–120). Studies by our group have demonstrated that the presence of activated platelets and increased PMC formation increases the rate of monocyte extravasation across the damaged BBB, and increased level of nonclassical proinflammatory monocytes correlated negatively with in people with CSVD and cognitive impairment (121, 122).

In addition to vascular disease and age-related comorbidities, many viruses are associated with increased inflammatory responses and risk of vascular damage, such as SARS-CoV-2, the causative agent of COVID-19 (123–125) and Human Immunodeficiency Virus (HIV) infection. Acute SARS-CoV-2 infection often results in increased levels of platelet activation (126, 127). Not only is platelet activation a biproduct of the inflammatory state caused by the infection, but it has been shown that platelets may actually drive the severe vascular inflammation observed in many patients (127, 128). For some patients, the aftereffects of SARS-CoV-2 infection do not dissipate after the expected two week period and can result in long COVID (129–131). One of the key signatures of long COVID is low-level, sustained inflammation that often results in the development of other autoimmune comorbidities such as mast cell activation syndrome, Systemic Lupus Erythematosus, Guillain-Barré syndrome, and rheumatoid arthritis (132–134). This sustained inflammation also commonly results in continued platelet activation, dysregulation of platelet function resulting in increased risk of vascular events, endotheliopathy, lung impairment, and induction of complement-mediated tissue damage (135–140). Long COVID also results in increased platelet leukocyte complexes, which, contribute to the sustained inflammatory environment (141–143).

Another condition where inflammation and platelet activation play a huge role is in Human Immunodeficiency Virus (HIV) infection, which has transitioned into a “chronic inflammatory condition” with the advent of antiretroviral therapy (ART) and has been shown to be associated with excessively activated platelets (144–146). ART has been shown to directly activate platelets via interruption of nitric oxide mediated platelet inhibition as well as result in decreased platelet-derived lipid production and increased platelet aggregation (147, 148). Since ART has significantly improved the lifespan of people with HIV, there is a significant increase in population of individuals ageing with ART controlled HIV resulting in increased incidence of inflammation and age associated comorbidities, often coined “inflammageing” (149–152). The comorbidities under the HIV-related inflammageing umbrella include cardiovascular disease, specifically atherosclerosis, neurological conditions, and osteoporosis (153–157). Each of these conditions is associated with increased platelet activation indicative of both the causative inflammation, as well as increased pathology (99, 158–160). A study by Knudsen et al. discovered that PLWH experience a three-fold increase in the risk of obstructive coronary atherosclerosis (161). As discussed previously, PLWH have increased levels of platelet activation which has been shown to directly correlate with increased risk of AS progression (162).There is also evidence that platelets can actually serve as a viral reservoir that can actively infect CD4^+^ T cells during complex formation (163). Consequently, increased levels of TGFβ in circulation as well as various tissues has been reported by several groups (164–170).

Outside of viral infections, it has also been shown that an increased number of activated circulating platelets are associated with an increased disease pathology and more severe prognosis in patients with various cancer types and metabolic conditions (171–176). Several studies have observed that in cancers with solid tumors, platelets have a protective function around the tumor site, meaning that they both physically and chemically inhibit immune elimination of the cancer cells which allows for tumor growth and enhanced metastasis (171, 177–183). Moreover, elevated plasma TGFβ was found to be associated with different types of cancers including breast cancer and carcinoma (184, 185).

Taken together, the pathogenic nature of platelet activation and subsequent increase in platelet derived TGFβ is evident in chronic inflammatory conditions and can exacerbate immune suppression as well as pathogenic fibrosis.

## 3 TGFβ in health and disease and the impact of platelet-derived TGFβ

### 3.1 Immunosuppressive function of TGFβ

#### 3.1.1 General overview of TGFβ

TGFβ is crucial for suppressing Th1 inflammatory responses and preventing inflammation-induced tissue damage. A study by Shull et al. showed that depleting TGFβ in mice led to the development of a lethal inflammatory disorder in which Th1 cells accumulated within key organs, ultimately resulting in organ damage due to uncontrolled IFN-γ secretion by the Th1 cells (186–188). The mechanism by which TGFβ prevents Th1 differentiation is via inhibition of the master regulator T-bet while also inhibiting IFN-γ secretion via the protein tyrosine phosphatase Src homology region 2 domain-containing phosphatase-1 (PTP Shp-1) (189, 190). TGFβ also induces an increase in IL-10 production which leads to increased suppression of inflammatory responses via Th1 cells (191–194) and the induction of TGFβ and IL-10 is beneficial in conditions like allergies or Lupus in which the inflammatory nature of Th1 cells drive the disease pathology.

#### 3.1.2 Platelet-derived TGFβ

As discussed previously, platelets have been shown to have immunosuppressive masking effects on cancer cells, leading to increased rate of metastasis. Specifically, platelet-derived TGFβ has been shown to directly increase rates of tumor cell extravasation as well as increased immunosuppressive cell polarization (195–198). Platelet-TGFβ also increases the rate of glucose consumption by cancer cells through activation of the AKT signaling pathway (196, 199). Increased glucose consumption correlates with higher rates of tumor cell proliferation, as well as increased migration and invasion (200, 201). Saito et al. showed that platelet-derived TGFβ induced Tissue factor expression in osteosarcoma cells using the SMAD3 pathway and aided in lung metastasis (202). In addition to tumor-cell specific effects, platelet-derived TGFβ has been shown to induce expansion and reprogramming of myeloid derived suppressor cells (MDSCs) and regulatory T cells (Tregs), thus directly contributing to the immunosuppressive milieu found within the tumor microenvironment (69, 197, 203).

Our group, among others, have demonstrated that in people living with HIV (PLWH), activated platelets form transient complexes with immune cells in circulation, especially monocytes (PMCs) (121, 163, 204–208). We posit that being in close proximity to platelets can increase the probability of these immune cells to be exposed to an increased bolus of platelet-derived immune molecules. In this regard we have shown that PMC-derived dendritic cells exhibit decreased ability to stimulate antiviral T cell response due to impaired antigen uptake and key cytokine secretion (209). As mentioned before, HIV infection, even when well-controlled on ART, is associated with an increase in TGFβ levels (164–166, 210). Our results indicate that, activated as well as not activated platelets from PLWH produce higher levels of TGFβ as compared with age, sex matched people living without HIV (PLWoH) (211). When monocytes are exposed to TGFβ at doses secreted by activated platelets, they downregulate functionally important surface markers associated with antiviral immune responses and antigen presentation (211). Moreover initial exposure to platelet-derived TGFβ induced PMC-derived dendritic cells to produce higher levels of endogenous TGFβ, thus exacerbating the immunosuppressive phenotype via autocrine and paracrine TGFβ signaling (24, 25, 211). Importantly, PMC-derived dendritic cells from PLWH were not able to stimulate a robust CD8^+^ T cell response compared to PLWoH (211). *In vitro* treatment of platelets with aspirin and clopidogrel was effective in PLWoH but failed mitigate the immunosuppressive effects of platelet derived TGFβ in PLWH (211).

These studies indicate that during homeostasis as well as in conditions where excessive Th1 responses underlie disease progression, TGFβ may have beneficial immunosuppressive functions. But this paradigm does not hold true for conditions that are associated with reduced or dysfunctional immune responses, for example cancers or HIV infection, where excessive levels of platelet derived TGFβ can lead to insufficient antiviral or antitumoral immune responses.

### 3.2 Wound healing function of TGFβ

#### 3.2.1 General overview of TGFβ

After injury, TGFβ is involved in each of the main stages of tissue repair. These stages include hemostasis and inflammation, proliferation, and ECM remodeling (212–214). TGFβ both recruits and polarizes various infiltrating cell types including neutrophils, mast cells, and monocytes throughout the 24 to 96 hours after injury (212, 215–220). While TGFβ aids in the initial immune infiltration of these cells, it simultaneously exerts its immunosuppressive functions to limit excessive inflammation through antagonism of other chemoattractants and polarization of immune cells (212, 216). The proliferative phase is comprised of three TGFβ-mediated phases including re-epithelialization, angiogenesis, and ECM synthesis (212, 221–224). Finally, healing comes to a resolution with the final stage known as remodeling. This process involves the apoptosis of the activated fibroblasts as well as endothelial cells, and a replacement of fibronectin and type III collagen with type I collagen turning the vascular, granulated tissue into an acellular and avascular scar (212, 225–229).

Throughout this process, a delicate balance between TGFβ-mediated epithelial to mesenchymal transition (EMT) and endothelial to mesenchymal transition (EndMT) as well as the reversal of EMT/EndMT play a crucial role in correct would healing (reviewed in (230, 231)). EMT is the main process that drives re-epithelialization involving the canonical TGFβ signaling pathway (212, 232). SMAD3 within this pathway is a key regulator for switching between TGFβ-suppressed cell growth under homeostasis and TGFβ-induced EMT following tissue injury (212, 233–235). During ECM remodeling, the overall population of fibroblasts begins to diverge with some differentiating into myofibroblasts while the other undergo apoptosis, thus marking the transition into the final stage of wound healing (212, 236–239). Endothelial cells also contribute to this process by differentiating into myofibroblasts via endothelial to mesenchymal transition (EndMT) (240, 241). EndMT leads to the loss of endothelial identity and the gain of mesenchymal traits such as contractility, motility, and cytoskeletal remodeling (242, 243).

As described above, TGFβ signaling is crucial for proper wound and injury repair, so, as expected, a disruption in these pathways results in improper, delayed, or decreased rates of healing. A clear example of this is the chronic development of open wounds in people with diabetes. Diabetes is associated with increased levels of serum or kidney localized TGFβ which often results in nephritis (244–246). Nephritis is the result of increased ECM production triggered by the increased level of TGFβ (244, 247–249), however, this is not replicated in vascular wound healing and repair. A set of key features of chronic wounds associated with diabetes is prolonged inflammation and decreased response to infection and repair stimuli (250, 251). Due to the chronic nature of diabetes associated inflammation, following injury, there is increased influx of neutrophils and macrophages, specifically inflammatory macrophages that secrete increased levels of proinflammatory cytokines and reactive oxygen species that limit the production and secretion of TGFβ (251–256). Hyperglycemia is also associated with a reduction in endothelial cell and fibroblast proliferation, and the fibroblasts that do differentiate into myofibroblasts, often exhibit reduced functionality, resulting in decreased angiogenesis and ECM production due to increased levels of TNFα which reduces the expression of TGFβ (257, 258).

#### 3.2.2 Platelet-derived TGFβ

Immediately following injury, subendothelial collagen is exposed to platelets, resulting in the activation, coagulation, and degranulation as described previously (41, 45, 259). TGFβ in platelet alpha granules is released upon degranulation (38, 47, 260). While it is well understood that platelet activation is a key step in the initiation of wound healing, the role of platelet-derived TGFβ in this process is less characterized. Instead, it has been investigated as potential therapeutic target to improve TGFβ signaling and wound repair, not just in diabetes, but in many conditions where chronic wounds are common.

Trials of topical application of TGFβ have demonstrated promising results for improving tensile strength and wound closure, supporting the idea that exogenous TGFβ could be a promising therapeutic avenue for improving healing (224, 261–263). With this in mind, platelet lysate and platelet-rich plasma (PRP) have both been discussed as options for personalized treatments to increase the success of wound repair due to their high concentration of platelet-derived TGFβ and platelet-derived growth factor (PDGF) (264–267). Studies by Liu et al. and Radithia et al. showed that, topical application of platelet rich plasma promoted diabetic wound healing through TGFβ expression, subsequent activation of the PI3K/AKT and TGFβ/SMAD pathways, and suppression of matrix metalloprotein 9 (265, 266). A study by Jee et al. discovered that, although not topically administered, due to high concentrations of platelet-derived TGFβ and PDGF, intralesional injections of autologous PRP resulted in in more efficient wound healing in canine cutaneous wounds (267).

In addition to PRP, platelet lysate (PL) is another therapeutic avenue that has been explored. PL, due to the additional lysing step during processing, results in increased immediate potency of growth factors while PRP releases platelet factors over a period of days to weeks (268). Barsotti et al. investigated the ability for allogeneic PL to be a therapeutic strategy for wound healing and tissue regeneration (264). They found that PL was sufficient to improve wound healing via beneficial effects on the various cell types involved throughout wound healing, specifically mentioning TGFβ as a leading factor contributing to this behavior, and, raise the point that allogeneic PL collected from healthy donors could minimize the effects of underlying health conditions such as those found in the patients receiving the PL treatment (264).

Interestingly, a study by Chong et al. demonstrated that, in their model, platelet-derived TGFβ was not directly involved in fibroblast recruitment and wound closure, but was instead crucial for enhancing keratinocyte proliferation and epidermal remodeling (269). This highlights a key issue, that, although it has been shown that platelet-derived TGFβ clearly enhances wound healing, the mechanism by which these results are achieved may be different across various conditions. A conclusive, universal treatment option for addressing platelet derived TGFβ insufficiency in impaired wound healing has not yet been determined and illuminate a key area for future research.

### 3.3 Profibrotic functions of TGFβ

#### 3.3.1 General overview of TGFβ

Just like a deficiency in TGFβ can impair wound healing, overaccumulation of TGFβ can result in excessive EMT or EndMT and subsequently incorrect or overactive wound healing, specifically in individuals living with chronic inflammatory conditions. TGFβ mediated fibrotic pathology has been observed in cardiovascular diseases including coronary artery disease, aortic stenosis, cardiac fibrosis pulmonary arterial hypertension, as well as other conditions across different tissues such as endometriosis, kidney fibrosis, cerebral cavernous malformations, hematologic malignancies and blood-brain barrier dysfunction (109, 242, 270–276).

Atherosclerosis is linked to increased EndMT and chronic inflammation. Damage or injury to the inner layers of an artery trigger plaque formation (100, 101). It is suspected that EndMT triggered by this initial injury may be the causative agent of AS progression (277, 278). It is important to note that throughout the stages of plaque formation, the role of TGFβ is dynamic. At the early stages of plaque formation, to avoid plaque disruption and potential embolism, TGFβ is crucial for stabilizing the plaque. Increased levels of TGFβ result in increased extracellular matrix deposition and fibrotic cap formation, thus stabilizing the plaque and preventing it from dislodging or rupturing (279–282). However, in conditions such as HIV in which the levels of TGFβ, both platelet-derived and otherwise, are consistently elevated, this beneficial stability quickly transitions into increased fibrotic behavior and disease pathology, demonstrating the dynamic nature of TGFβ in AS progression (210, 211, 283–286).

#### 3.3.2 Platelet-derived TGFβ

Platelets, are associated with the progression of EndMT and pervasive fibrosis, specifically through the production of platelet-derived TGFβ (287–289). Meyer et al. have shown that following constriction of the transverse aorta, increased levels of platelet-derived TGFβ actively contributed to increased disease pathology including hypertrophy, systolic dysfunction, and fibrosis (35). Platelet-derived TGFβ was found to interact with fibroblasts and promote Angiotensin II induced atrial fibrillation (290). Increased levels of platelet-derived TGFβ have been found to be directly related to progression of HIV-associated fibrotic conditions (291–293). A study by our group found that macrophages differentiated from platelet complexed monocytes secreted higher levels of TGFβ compared to platelet null conditions, and PMCs from HIV^+^AS^+^ individuals exhibited increased TGFβ expression when compared to groups without HIV infection or without AS (294). They also found that, PMC-derived macrophages induced EndMT by downregulating endothelial markers and upregulating mesenchymal markers in *in vitro* co-cultures, when compared to non-PMC-derived macrophages. These studies altogether demonstrate a key link between platelet-derived TGFβ and EndMT and fibrosis during cardiovascular disease.

Platelet-derived TGFβ has been shown to enhance the rates of epithelial-mesenchymal transition (EMT), allowing for increased invasion by tumor cells in osteosarcoma, hepatocellular carcinoma and gastric carcinoma (178, 295–297). Using two mouse models of renal fibrosis, it was shown that platelet-derived TGFβ induced EMT in renal proximal tubular epithelial cells and these fibrotic effects were rescued by selective clocking of the TGFβ receptor (298). Similar results were seen in another study, where depleting platelet-derived TGFβ, decreased liver fibrosis (299).

Overall, these findings indicate that during chronic inflammatory conditions, excessive platelet activation and subsequent increase in platelet-derived TGFβ can underlie uncontrolled EMT/EndMT and thus result in pathogenic fibrosis.

## 4 Therapeutic approaches to inhibit platelet activation and platelet-derived TGFβ

### 4.1 Overview of therapeutic approaches

There are multiple potential approaches to regulate TGFβ expression: 1) TGFβ inhibitors, 2) TGFβ receptor inhibitors, 3) anti platelet drugs (APD). The first two categories would inhibit overall expression of TGFβ while APD is an FDA approved approach to curb platelet activation including all platelet-origin immune molecules and as such is not TGFβ-specific inhibitor. In addition, there are conditions where an increase in TGFβ level may be desirable and therapeutic approaches to address those are discussed in section 3.2.2. There are multiple excellent reviews on TGFβ and TGFβ receptor inhibitors, which fall under the categories of monoclonal antibodies (for example Fresolimumab) or small molecule inhibitors (for example Galunisertib, Vectosertib) and are being investigated through phase I and II clinical trials against cancers and fibrosis (9, 300, 301). Combination therapies targeting TGFβ and PD-L1 are also underway to overcome TGFβ driven immune exclusion (302, 303). While these approaches appear to be promising they are associated with significant systemic toxicity, cardiovascular toxicity, impaired wound healing and triggering of autoimmune or inflammatory responses.

In this regard, APD appears to be a more viable approach since, it would affect only platelet-specific TGFβ production. APD is often prescribed as a preventative or therapeutic against vascular diseases and consists of cycloogenase-1 (COX-1) inhibitors (Aspirin), ADP P_2_Y_12_ receptor antagonists (Clopidogrel, Prasugrel, Ticagrelor), or platelet glycoprotein IIb/IIIa inhibitors (abciximab or tirofiban), although the latter is primarily prescribed in more severe cases after occurrence of ischemic events (304–310). Aspirin, inhibits the COX-1 enzyme which is upstream of thromboxane A_2_ synthesis, thus preventing a crucial step in the platelet coagulation cascade (311). P_2_Y_12_ inhibitors function by preventing the binding of adenosine diphosphate (ADP) to its cognate platelet P2Y_12_ receptor and thereby preventing platelet activation by disrupting the formation of the GP IIb/IIIa complex (312, 313). GP IIb/IIIa inhibitors function by preventing the final step in platelet aggregation and thrombus formation (309, 314).

The ability of these drugs to reduce clot formation and vascular disease has been well documented. Since platelets house a wide array of immunogenic molecules, it is important to also consider the potential implications of APD usage in other chronic inflammatory conditions where platelets are activated. Hence in the following sections we are focusing on APD in viral infections, cancers and fibrosis and what is currently known about its effects on platelet-derived TGFβ.

### 4.2 Therapeutic potential of APD in chronic inflammatory conditions

#### 4.2.1 APD in viral infections

Many groups have investigated the impact of APD therapy on disease severity during SARS-CoV-2 infection (315). The results of these studies are rather inconclusive with some saying there is little effect of Aspirin or P_2_Y_12_ receptor antagonists in reducing mortality and disease severity (316, 317) with others noting that there is an association between combined APD treatments and a decreased need for mechanical ventilation and all-cause mortality in patients with COVID-19 (318, 319). There is evidence that patients who had been regularly taking APD prior to infection had reduced rates of mortality (320). On the other hand, APD use during severe SARS-CoV-2 infection also resulted in platelet hypo reactivity, both limiting their coagulative functions and also their ability to regulate immune responses, specifically platelet-mediated neutrophil responses (321, 322).

Similar to SARS-CoV-2, the usage of APD therapies for people living with HIV have been investigated for both reducing overall platelet activation and inflammation, as well as studying the ability for APD to potentially improve responses to ART. ART such as ritonavir and cobicistat are known to improve plasma concentrations of other ART drugs when given in combination through the inhibition of cytochrome p450 (323, 324). Cytochrome p450 is a family of enzymes responsible for hepatic metabolism of many drugs, including Aspirin and Clopidogrel (325). So, while ART inhibition of cytochrome p450 improves the efficacy of several other ART drugs, it directly reduces the ability for APD to be metabolized and reduce inflammation and platelet activation. Clopidogrel, while effective in reducing platelet activation in people living without HIV, was only about 50% effective in people living with ART controlled HIV (324). In a clinical trial conducted by Marcantoni et al, Clopidogrel successfully reduced endothelial activation due to platelet activation in patients with ART controlled HIV, however, they found that Aspirin had no effect (323). Even at clinically relevant high dosages of 300mg (326), Aspirin had little to no effect on HIV associated inflammation (327). Newer APD, such as Ticagrelor is not a prodrug like Aspirin, Clopidogrel, meaning that the cytochrome p450 cleavage event is not required (328, 329). These findings indicate that APD at the clinically prescribed dosage may not be as efficient in people with HIV as compared to uninfected individuals and it is possible that Ticagrelor could be a potential avenue to explore for PLWH on ART.

#### 4.2.2 APD in cancers and fibrosis

Aspirin was shown to be beneficial in colorectal and pancreatic cancers by limiting the production of platelet-derived oncogenes, specifically c-MYC, and the secretion of prostaglandin E2 (PGE2) (330–334). Additionally, GP IIb/IIIa inhibitors have also been discussed as having a potential benefit in reducing platelet-mediated metastasis due to disrupting the ability for platelets to bind to circulating leukocytes and cancer cells (335–338). Mouse studies have demonstrated that inhibition of the GP IIb/IIIa complex or the cognate ligands on tumor cells decreases the rate of metastasis to that seen in models where platelets have been depleted entirely, thus showing a strong link between activated platelets and worsened prognosis (171, 335, 339, 340). However, as described above, GP IIb/IIIa inhibitors are not feasible over extended periods of time due to risk of immune thrombocytopenia and severe bleeding. A study by Toragall et al. discussed the potential efficacy of a new antiplatelet drug that targets the thromboxane A_2_-prostanoid receptor which would prevent the interactions of platelets to circulating breast cancer cells (341). This study demonstrated a significant reduction in platelet/tumor cell complexes and a reduced incidence of metastasis (341).

Several mouse and rat model studies have shown that APD has beneficial effects in liver, pulmonary and cardiac fibrosis (342–347). With regards to humans, in a prospective cross sectional study, use of APD was found to be protective against liver fibrosis (348). Low dose aspirin was found to reduce liver fibrosis progression after transplantation (349), in people with nonalcoholic fatty liver disease (350) as well as in older adults (351).

### 4.3 APD and platelet-derived TGFβ

As described above the potential benefit of APD in reducing the comorbidity associated with viral infections, cancers as well as fibrotic pathology is unambiguous, but none of these studies have actually investigated the molecular mechanisms by which APD confers the protection and certainly these studies have not addressed the status of circulating or tissue levels of TGFβ.

Extensive literature search yielded only a few studies in this regard. Jayaram et al. conducted a clinical trial where healthy individuals were enrolled and were treated with low dose aspirin for 14 days (352). The levels of TGFβ and other growth factors produced by ex vivo activated platelets before and after aspirin treatment were measured. They found that after aspirin treatment significantly lowered the levels of TGFβ, with average TGFβ concentration of 40pg/ml before aspirin as compared to 7pg/ml after aspirin (352). Another study by Zhang et al. compared the TGFβ produced by platelets from people with diabetes mellitus who were not taking aspirin as compared to people with diabetes who were taking aspirin (353). This study also included a group of healthy individuals not taking aspirin. They found that both the groups who had diabetes produced lower levels of TGFβ (average TGFβ concentration of 750pg/ml) as compared to the healthy group (average TGFβ concentration of 1000pg/ml), however aspirin had no effect on the amount of TGFβ produced (353). Anitua et al. found that aspirin dosage of 100mg/d did not affect the levels of TGFβ (354). Similar findings were reported by Smith et al, where people taking 80mg/d aspirin had same levels of TGFβ as compared people not taking APD. Only those who were on dual antiplatelet therapy (aspirin and clopidogrel) showed significant reduction (355). Our group has reported similar results with ex vivo treatment of platelets with aspirin and clopidogrel, where in platelets from people living with HIV, only clopidogrel treatment led to reduction in TGFβ levels (211). Feng at al measured the levels of TGFβ after a combination treatment of indobufen (a reversible Cox inhibitor) and clopidogrel after percutaneous transluminal angioplasty of arteriovenous fistula and compared the levels with those only treated with clopidogrel (356). They reported plasma TGFβ levels of average 80pg/ml in both the groups, but since the study design did not allow the inclusion of a no APD group, it is not possible to compare the levels of TGFβ with and without clopidogrel (356).

Overall, these studies indicate that APD seems to improve disease outcomes in SARS-CoV-2, HIV infections or cancers or profibrotic conditions. Low dose aspirin is potent enough to reduce thrombosis and platelet aggregation but may not be sufficient to significantly reduce platelet-derived TGFβ levels. In this regard P2Y12 inhibitors, may be more useful, but at the present moment there is not sufficient data to conclusively state how efficacious this class of APD is. This matter is further complicated for conditions such as diabetes (357, 358) or HIV infection where the clinically prescribed dosages of aspirin or clopidogrel may not be as effective as in individuals without these comorbidities. Lastly, usage of APD has been associated with bleeding complications, particularly intracranial and gastrointestinal hemorrhage. These adverse effects needs to be considered before prescribing APD.

## 5 Discussion

It is crucial to understand that, although TGFβ is canonically associated with homeostatic immunoregulation, the directionality of whether platelet-derived TGFβ is beneficial or harmful are incredibly dependent on the disease specific immune context. In the presence of chronic inflammation, this normally homeostatic behavior can result in increased disease-associated comorbidities (**Figure 1**). Due to this, an ideal therapeutic approach would be to partially inhibit platelet derived TGFβ without disturbing homeostasis and wound healing but at the same time, curbing the unintended pathogenic effects.

**Figure 1:**
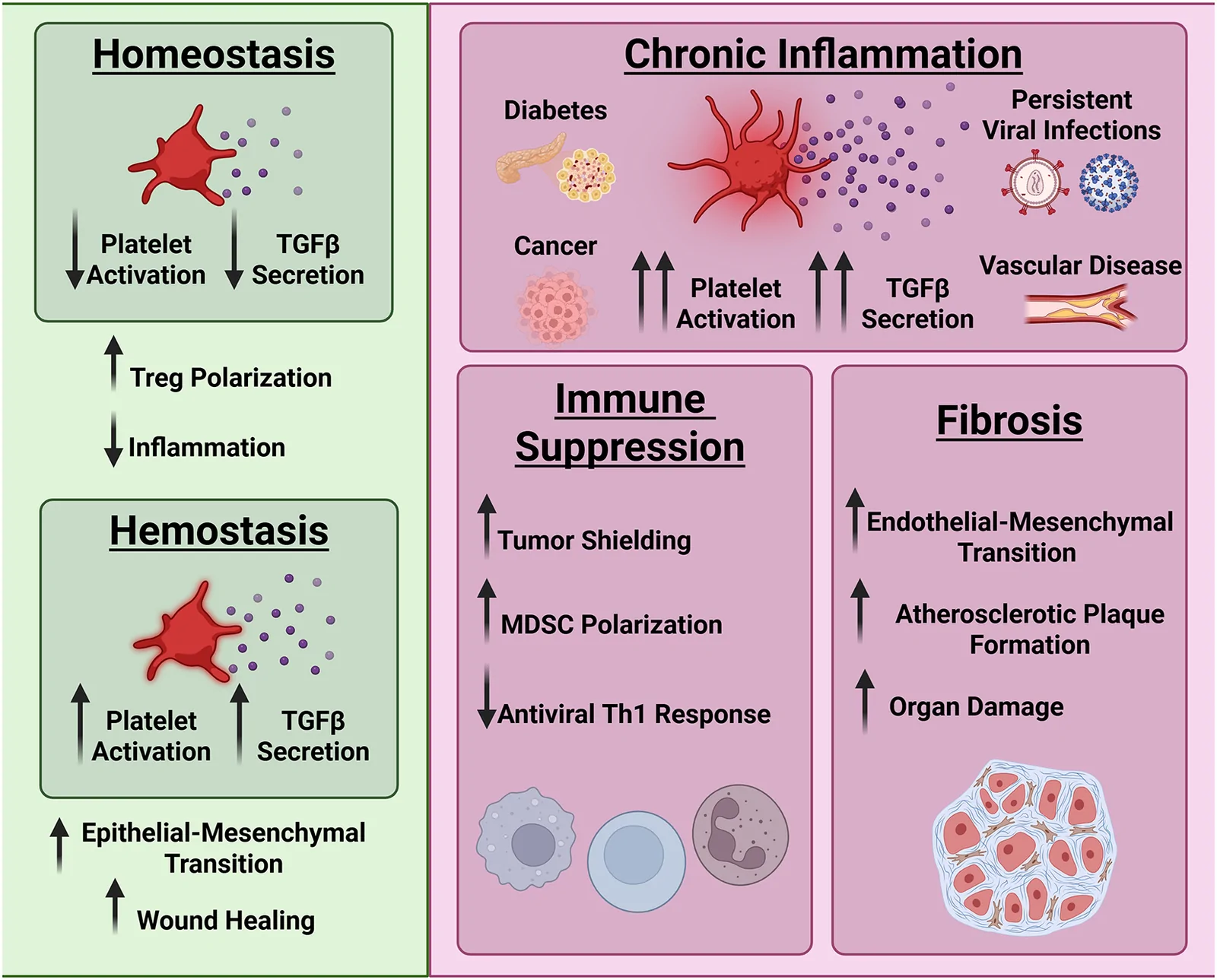
Under normal conditions, platelets exist at a baseline state of low activity and low-level TGFβ secretion. This tonic TGFβ signaling mediates immune homeostasis through the polarization of regulatory T lymphocytes and dampening of inflammatory responses. Following tissue injury, platelets become transiently activated, leading to increased secretion of TGFβ, which initiates epithelial to mesenchymal transition, ultimately resulting in resolution of the injury and wound healing. In this regard, temporary increases in platelet TGFβ play a protective role. However, in chronic inflammatory conditions that are associated with persistent platelets activation, such as HIV and SARS-CoV-2, diabetes, vascular disease, cancers, etc., TGFβ can be pathogenic. Elevated levels of TGFβ during these conditions can result in aberrant immune suppression leading to increased tumor shielding and reduced antiviral responses, as well as excessive vascular fibrosis, and eventual tissue and organ damage. This highlights that whether TGFβ plays a pathogenic or pro-homeostatic role is dependent on the disease context, which is specifically relevant in conditions associated with excessive, long term platelet activation. Created in BioRender. Sutton, K. (2026) https://BioRender.com/m5fjqd1.

The complication with untangling the contributions of platelet-derived TGFβ to disease severity and pathology, and the ability to mitigate it, lies in four major gaps in the field. First, chronic inflammatory conditions including HIV, CVD, Long Covid, diabetes, and the other conditions discussed above often have complex multi-organ system implications. This wide-spread inflammation or disease presence can make it challenging to uncover the true effects of a single molecule on the progression of symptoms or pathology, especially when a baseline was not determined prior to the onset of the condition.

Secondly, while the effects of APD on the immune system both in the presence and absence of chronic inflammatory conditions have been explored by several groups, these results are often contradictory or inconclusive. The effects of short-term APD administration to improve the immune response to acute viral infections are vastly different than sustained use to mitigate vascular symptoms resulting from a chronic condition. With that said, there are still very few studies that have shown the effects of long-term APD use on the immune system or on platelet associated immune function. Hence to understand how APD use can mitigate pathogenic effects of platelet-derived TGFβ during chronic inflammatory conditions, this gap would first need to be addressed.

Third, although platelets are now being appreciated as highly immunomodulatory cells in addition to their role in hemostasis and wound healing, the field is still relatively new. However, this means that there is exciting unexplored potential for harnessing platelet-derived products for powerful therapeutic strategies. TGFβ is a promising target for immune modulation but understanding how other platelet-derived molecules interact with both TGFβ, and the greater immune environment is crucial for gaining a deeper mechanistic understanding of how platelet-derived TGFβ responds to and behaves in both homeostatic and disease conditions.

Lastly, there are technical difficulties in accurately measuring the levels of TGFβ in circulation and at different tissue sites. As discussed in the introduction, there are multiple different ways in which latent TGFβ can be activated, which makes it nearly impossible to determine what the concentration of active TGFβ would be at the site of injury. Measurement of TGFβ in serum or plasma is complicated by the fact that venipuncture or the process of plasma/serum isolation can activate platelets and they can degranulate resulting in overestimation of the circulating levels of TGFβ (359, 360). Moreover, the most commonly used assays to measure TGFβ is ELISA and many kits utilize acid based activation of latent TGFβ, which does not necessarily reflect the amount to active TGFβ *in vivo*. This is reflected in the human studies discussed in section 4.3, which report widely different ranges on circulating TGFβ. All of these limitations make it extremely difficult to define the TGFβ concentration thresholds where its effects switch from beneficial to pathogenic in a disease dependent manner.

While there is much, we still do not know about platelets and platelet-derived TGFβ in modulating immune function and disease pathology, it provides a valuable insight into how longstanding immune paradigms can and should be challenged depending on the context in which they are being investigated.
